# Effect of Catnip Charcoal on the* In Vivo* Pharmacokinetics of the Main Alkaloids of Rhizoma Coptidis

**DOI:** 10.1155/2016/3532159

**Published:** 2016-05-25

**Authors:** Yanfei He, Siyu Chen, Hai Yu, Long Zhu, Yayun Liu, Chunyang Han, Cuiyan Liu

**Affiliations:** College of Animal Science and Technology, Anhui Agricultural University, 130 Changjiang West Road, Hefei, Anhui 230036, China

## Abstract

This study aims to explore the effect of catnip* Nepeta cataria* (CNC) charcoal on the pharmacokinetics of the main alkaloids of Rhizoma Coptidis* in vivo*. Twenty-four rabbits were randomly divided into four groups and given oral administration of an aqueous extract of Rhizoma Coptidis (RCAE), RCAE plus CNC, RCAE plus activated carbon (AC), or distilled water, respectively. Plasma samples were collected after administration. The concentrations of berberine, coptisine, palmatine, and epiberberine in plasma were measured by high-performance liquid chromatography (HPLC). The pharmacokinetics data were calculated using pharmacokinetic DAS 2.0 software. The results showed that the area under the concentration-time curve (AUC) of berberine increased, while the AUC of coptisine, palmatine, and epiberberine decreased in the rabbits that received RCAE plus CNC. Meanwhile, the AUC of berberine, coptisine, palmatine, and epiberberine decreased in the group given RCAE plus AC. The difference of main pharmacokinetics parameters among the four groups was significant (*P* < 0.05). This study showed that CNC improved the bioavailability of berberine in comparison to AC and prolonged its release in comparison to RCAE alone. However, it decreased the bioavailability of coptisine, palmatine, and epiberberine. In comparison, AC uniformly declined the bioavailability of berberine, coptisine, palmatine, and epiberberine.

## 1. Introduction

Herbal charcoals have been used traditionally in Chinese medicine for many years, being one of the most characteristic processing methods of Chinese herbal medicines with the purpose of changing the herbal nature, enhancing the astringency, hemostasis, and antidiarrheal activities, and also reducing toxicity of some herbals [1, 2]. The catnip* Nepeta cataria* (CNC) charcoal is typically made from cut pieces of CNC, which are carbonized until coke-black on a strong fire. Catnip* Nepeta cataria* (CNC) charcoal has been shown to exhibit better effects than the noncharcoal form in the treatment of hematochezia, metrorrhagia, and postpartum anemic fainting [3]. Notably, although in charcoal form, various charcoals of Chinese herbs partially retain the inherent nature of the raw herbal [4].

Pharmacological research has indicated that the charcoal form of Chinese herbal medicines could enhance the astringency, hemostasis, and antidiarrheal activity of herbs due to the absorption and astringency of activated carbon (AC), which is generated during the processing of charcoals [5, 6]. It was unclear, however, whether the carbonized herbs subsequently absorbed the active components of other herbals when used in combination, thus decreasing their therapeutic effects due to nonselective absorption of AC. In addition, Mullins et al. found that AC could accelerate the excretion of other drugs from the body and decrease the bioavailability of some drugs due to the interruption of drug recirculation following reabsorption from the gastrointestinal tract or the promotion of vasoconstriction of the capillaries in the intestinal wall [7]. In summary, no common consensus has been reached with regard to the mechanisms of carbonized Chinese herbal medicines and their effects on other drugs taken concomitantly.

**Figure 1:**
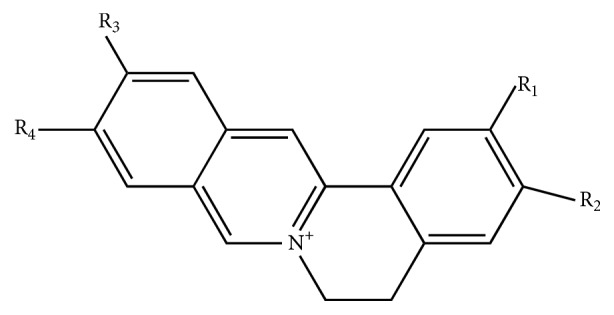
The molecular structure of berberine, epiberberine, coptisine, and palmatine. Note: berberine (R_1_-R_2_ = –O–CH_3_–O–; R_3_ = –OCH_3_; R_4_ = –OCH_3_); epiberberine (R_1_ = –OCH_3_; R_2_ = –OCH_3_; R_3_-R_4_ = –O–CH_3_–O–); coptisine (R_1_-R_2_ = –O–CH_3_–O–; R_3_-R_4_ = –O–CH_3_–O–); palmatine (R_1_ = –OCH_3_; R_2_ = –OCH_3_; R_3_ = –OCH_3_; R_4_ = –OCH_3_).

*Nepeta cataria* has an acrid and bitter taste. From a traditional Chinese medicinal perspective, it is slightly warm in nature and often used to expel pathogenic wind from the body surface. Clinically, it may be used to treat exanthema and as a hemostatic. On the other hand, Rhizoma Coptidis (RC) has been used in traditional Chinese medicine to clear heat, purge intense heat, and dry dampness and it may also be used in detoxification.* Nepeta cataria* and RC have been used together to clear “heat evil,” eliminate wind, and relieve liver conditions, as a part of the Jingjielianqiao decoction [8]. The purpose of this study was to clarify the effect of carbonized Chinese herbal medicines on the absorption of other drugs taken concomitantly. As such, to provide a basis for the clinical application of carbonized Chinese herbal medicines, the effect of CNC on the pharmacokinetics of berberine, coptisine, palmatine, and epiberberine, which are the main alkaloids in RC (showed in Figure 1), was investigated.

## 2. Materials and Methods

### 2.1. Agilent 1100 Series HPLC System

The Agilent 1100 Series LC consists of Agilent 1100 Series Quaternary Pump (G1311A), Agilent 1100 Series Autosampler (G1313A), Agilent 1100 Series Thermostatted Column Compartment (G1316A), Agilent 1100 Series Vacuum Degasser (G1379A), and Agilent 1100 Series variable wavelength UV detector (G1314A).

### 2.2. Herbal Medicines and Reagents

RC and CNC were purchased from Hefei Lejia Herbal Pieces Co. Ltd. (Anhui, China) and authenticated by Professor Shunxin Guo (Chinese Academy of Medical Science, Peking Union Medical College Institution of Medicinal Plant Development) in accordance with the* Chinese Pharmacopoeia*, 2010 edition. Medicinal AC was purchased from Sinopharm Chemical Reagent Co. Ltd. (China). Berberine, coptisine, palmatine, and epiberberine were purchased from the National Institutes for Food and Drug Control (China). High-performance liquid chromatography (HPLC) grade methanol and acetonitrile were provided by J. T. Baker Co. Ltd. (USA). Potassium dihydrogen phosphate and sodium lauryl sulfate were obtained from Anaqua Chemicals Supply (USA).

### 2.3. Animals

Twenty-four clean-grade adult male New Zealand rabbits (scxk (Shandong) 2014-0006) weighing 3.1 ± 0.6 kg were purchased from Jinan Jinfeng Experimental Animal Co. Ltd. Animals were treated humanely according to the National Research Council's guidelines.

### 2.4. Preparation of Stock Solutions and Herbal Medicines

#### 2.4.1. Preparation of Standardized Solution

Stock solutions were prepared by dissolving the accurately weighed four standard reference compounds in methanol (28 *μ*g/mL for coptisine, 20 *μ*g/mL for epiberberine, 11 *μ*g/mL for palmatine, and 28 *μ*g/mL for berberine).

#### 2.4.2. Preparation of Aqueous Extract of RC (RCAE)

Fifty g RC was soaked in 600 mL water for 30 min and then boiled over a strong flame prior to simmering for 30 min and the decoction liquid was collected. The remaining herbal residue was mixed with 300 mL water and boiled for a second time. After filtration, the two filtrates were mixed and concentrated to 500 mL to attain a final concentration of 0.1 gram of raw herb in 1 mL (0.1 g/mL) using a rotary evaporator at 45°C. Then they were divided into three equal parts (a, b, and c): part b was mixed with CNC at the ratio of 0.5 percent, and part c was mixed with AC at the ratio of 0.5 percent.

#### 2.4.3. Preparation of Powdered CNC and AC

CNC and AC were pulverized and sieved using 80-mesh and 120-mesh strainers, respectively. The resulting fine powders were kept for use in this study.

### 2.5. HPLC Analysis for Charred* Nepeta cataria* and* Nepeta cataria*

According to the stipulation of Chinese Pharmacopoeia (2015), the condition of HPLC was set and detecting solution was preparated. A ZOBAX C18 chromatography column (250 mm × 4.6 mm, 5 *μ*m) was used in this study. The mobile phase was a mixture of water (A) and methol (B). The elution of p-menthone was performed using an isocratic method of 80% A. The flow rate was set at 0.8 mL/min. The injection volume was 10 *μ*L, temperature of the column oven was set at *T* = 25°C during all experiment, and the wavelength for detection was 252 nm. The reference solution of p-menthone was prepared by dissolving the accurately weighed p-menthone in methanol.

### 2.6. Establishment of HPLC Method for Detecting Four Alkaloids in Rhizoma Coptidis

A ZOBAX C18 (250 mm × 4.6 mm, 5 *μ*m) chromatography column was used in this study. The mobile phase consisted of water containing 0.05 mol/L potassium dihydrogen phosphate (A) and acetonitrile (B) at a ratio of 50 : 50 (v/v). The injection volume was 10 *μ*L, the flow rate was 0.8 mL/min, the column temperature was 25°C, and the wavelength for detection was 345 nm.

### 2.7. HPLC Method Validation

The specificity was tested by comparison of the plasma sample to blank rabbit plasma and blank plasma spiked with the four analytes using established HPLC methods to observe the interference from endogenous substances contained in the analyte. Six samples were tested for specificity.

Calibration curves were prepared using standard plasma samples with different concentrations of the four analytes, the standard concentrations of berberine were 7.0, 17.5, 35, 70, 100, and 140 ng/mL, the standard concentrations of coptisine were 1.4, 8.75, 17.5, 35, 70, and 140 ng/mL, the standard concentrations of palmatine were 13.5, 27, 56.25, 112.5, 225, and 550 ng/mL, and the standard concentrations of epiberberine were 5, 12.5, 25, 50, 80, and 100 ng/mL. The peak area of the analyte was set as the vertical coordinate and the concentration of the analyte was set along the *x*-axis.

The precision and accuracy were evaluated by assaying six sample replicates with low, medium, and high concentrations during a single day and measuring six sample replicates with low, medium, and high concentrations once a day for five days. The precision was measured by the relative standard deviation (RSD) and the accuracy was described by the relative error (RE).

The extraction recovery and matrix effects of the four analytes were determined at three levels with six replicates. The extraction recoveries were evaluated by comparing the peak area obtained from the plasma sample spiked before extraction with the plasma sample spiked after extraction. The matrix effect was investigated by comparing the peak area of the analyte added to the preextracted plasma from untreated rats with that of the analyte dissolved in matrix component-free reconstitution solvent.

### 2.8. Pharmacokinetics Experiments

Twenty-four New Zealand rabbits were randomly divided into four groups, labeled Group A, Group B, Group C, and Group D. Group A received RCAE orally, Group B received RCAE and CNC orally, Group C received RCAE and AC orally, and Group D was administrated distilled water orally. The dosage of Groups A, B, and C was 15 mL/kg. All rabbits were fasted 24 h before administration and had free access to water. Before and immediately after oral treatment, rabbit blood samples (2.0 mL) were obtained from the auricular vein and samples were subsequently taken 0.25, 0.5, 0.75, 1, 1.5, 2, 2.5, 3, 4, 6, 8, 10, 12, 14, 18, 24, 36, and 72 h after administration. The blood samples were immediately heparinized and centrifuged at 4000 rpm for 10 min at 4°C. Prepared plasma samples were stored at −20°C until analysis. All of the pharmacokinetic parameters were processed by noncompartmental analysis using DAS 2.0 software (Mathematical Pharmacology Professional Committee of China, Shanghai, China) and all animal studies were performed according to the* Guide for the Care and Use of Laboratory Animals*.

### 2.9. Plasma Sample Preparation

Plasma samples (200 *μ*L) were placed in Eppendorf tubes and extracted with 0.8 mL acetonitrile by vortex mixing for 3 min and ultrasonic extraction for 30 min. After centrifugation at 12000 rpm for 10 min, the supernatant (0.8 mL) was pipette-transferred to another Eppendorf tube. Then, the residue was extracted via the same procedure for a second time. The supernatant was combined for each sample and evaporated to dryness under nitrogen at 45°C. The residue was redissolved in 80 *μ*L of the HPLC mobile phase and the solution was filtrated through a microporous filter membrane (0.22 *μ*m) prior to analysis. All samples were measured within a week. Interday precision and accuracy of the assay reached the standard of quantitative analysis, and the standard samples in low, medium, and high concentration of four analyses were measured to assure the accuracy every day.

### 2.10. Statistical Analysis

Data were represented in the mean ± standard deviation of the mean. Comparisons between different groups were carried out by Turkey's test. The level of significance was set at *P* < 0.05. SPSS software (version 19.0, IBM, Inc., USA) was used in statistical analysis.

## 3. Results

### 3.1. HPLC Analysis for Charred* Nepeta cataria* and* Nepeta cataria*

**Figure 2:**
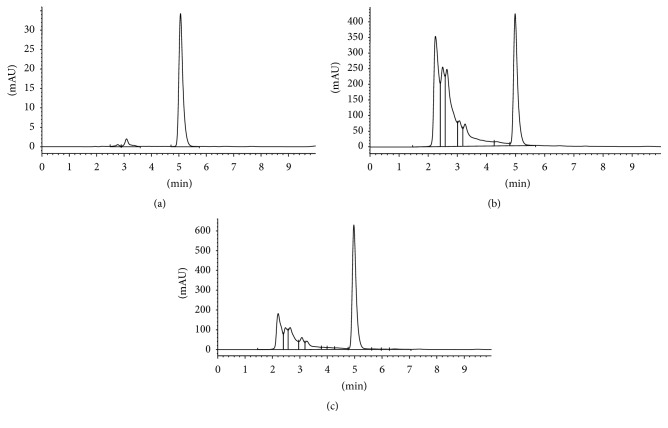
(a) shows the HPLC image of menthone, (b) shows the HPLC image of charred* Nepeta cataria*, and (c) shows the HPLC image of* Nepeta cataria*.

The retention time of p-menthone was 5.037 min under the stipulated HPLC condition, and the calibration curve was *y* = 12135*x* + 641.5 (*r* = 0.9999), as shown in Figure 2; (a) showed the HPLC image of p-menthone, (b) showed the HPLC image of charred* Nepeta cataria*, and (c) showed the HPLC image of* Nepeta cataria*. The results of the accuracy, precision, and extraction recovery showed that the extraction recovery was more than 90%, and both the RSD and reproducibility met the measure requirements. The results showed that the content of p-menthone of* Nepeta cataria* was 0.96 mg/g which was higher than 0.08% stipulated in* Chinese Pharmacopoeia* (2015) and the content of p-menthone of charred* Nepeta cataria* was 0.43 mg/g.

### 3.2. Specificity of the HPLC Method

**Figure 3:**
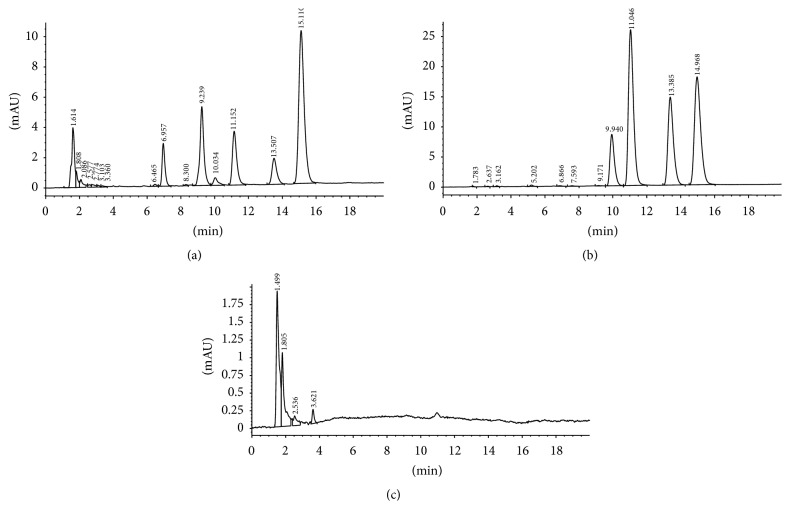
HPLC chromatogram of a plasma sample after administration (a), blank plasma spiked with the four analytes (b), and blank rabbit plasma (c).

The retention times of berberine, coptisine, palmatine, and epiberberine were 9.94, 11.10, 13.501, and 14.99 min, respectively. As shown in Figure 3, (a) showed the HPLC trace for the plasma sample after administration of RC, (b) showed the blank plasma spiked with the four analytes, and (c) showed the trace for blank plasma. There was no obvious interference from endogenous substances contained in the analytes according to the HPLC trace of blank rabbit plasma, blank plasma spiked with the four analytes, and the plasma sample after administration.

### 3.3. Calibration Curves

The standard curves of the four analytes all exhibited good linearity and good coefficients of correlation (*r* > 0.993). The limit of quantitation was appropriate for the quantitative detection of the four analytes in the plasma samples. The linear ranges, regression equations, and correlation coefficients were shown in Table 1.

### 3.4. Accuracy, Precision, and Extraction Replicates

The results of the specificity of the HPLC method showed that the matrix effect of the plasma taken from rabbits in the control group would not disturb measurement of the four alkaloids. The results of the accuracy, precision, and extraction recovery showed that the extraction recovery was more than 90%, and both the RSD and reproducibility met the measure requirements. All results showed that the HPLC method was reliable (Tables 2 and 3).

### 3.5. Concentration-Time Profile

#### 3.5.1. Concentration-Time Profile of Berberine in Plasma

**Figure 4:**
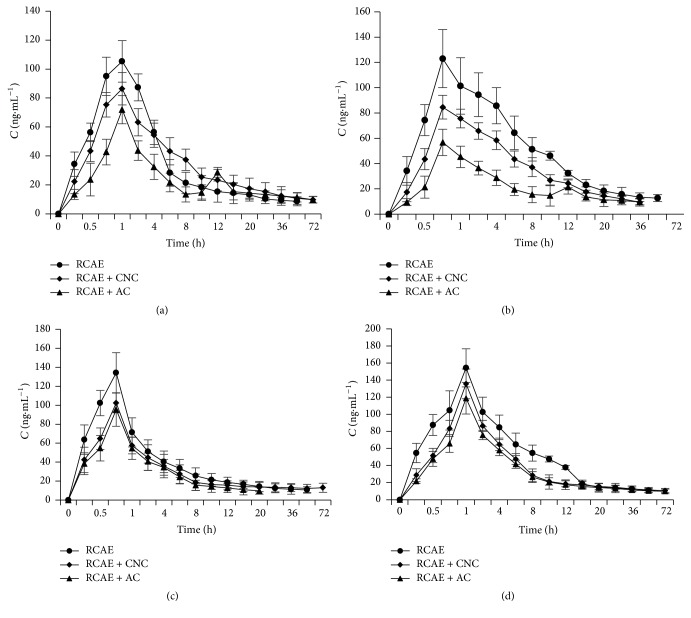
Concentration-time profile in plasma for berberine (a), coptisine (b), palmatine (c), and epiberberine (d).

Figure 4(a) showed the concentration changes of berberine in plasma extracted from rabbits in Groups A–C. The concentration of plasma berberine in Group A was higher than in Group B from the point of administration to 2 h after administration. However, at 4 h after administration, the concentration in Group B exceeded that of Group A and this trend continued up until 72 h after administration. Meanwhile, the concentration of plasma berberine in Group C was lower than in Groups A and B, although the berberine concentration of Group C did increase again at 10 h after administration, showing a second peak in the concentration profile at 12 h after administration.

#### 3.5.2. Concentration-Time Profile of Coptisine in Plasma

Figure 4(b) showed the concentration changes for coptisine in plasma for Groups A–C, indicating that the concentration in Group B was lower than in Group A during the study, while the concentration in Group C was lower than in Groups A and B. Again, the profile for Group C showed a second peak at 12 h after administration.

#### 3.5.3. Concentration-Time Profile of Palmatine in Plasma

Figure 4(c) showed the concentration changes of plasma palmatine in Groups A–C. As can be seen from the figure, the concentration of plasma palmatine in Group B was lower than in Group A during the study, but the concentration of plasma palmatine in Group C was lower than in Group B. However, the difference between Groups B and C was not significant.

#### 3.5.4. Concentration-Time Profile of Epiberberine in Plasma

Figure 4(d) showed the concentration changes of epiberberine in plasma for Groups A–C. Akin to the results for palmatine, the concentration of plasma epiberberine in Group B was lower than in Group A during the study, while the concentration of plasma epiberberine in Group C was lower than in Group B. Again, the difference between Groups B and C was not significant.

### 3.6. Maximum Plasma Concentration, Time to Reach the Maximum Concentration, Area under Curve, and Drug Half-Life

**Figure 5:**
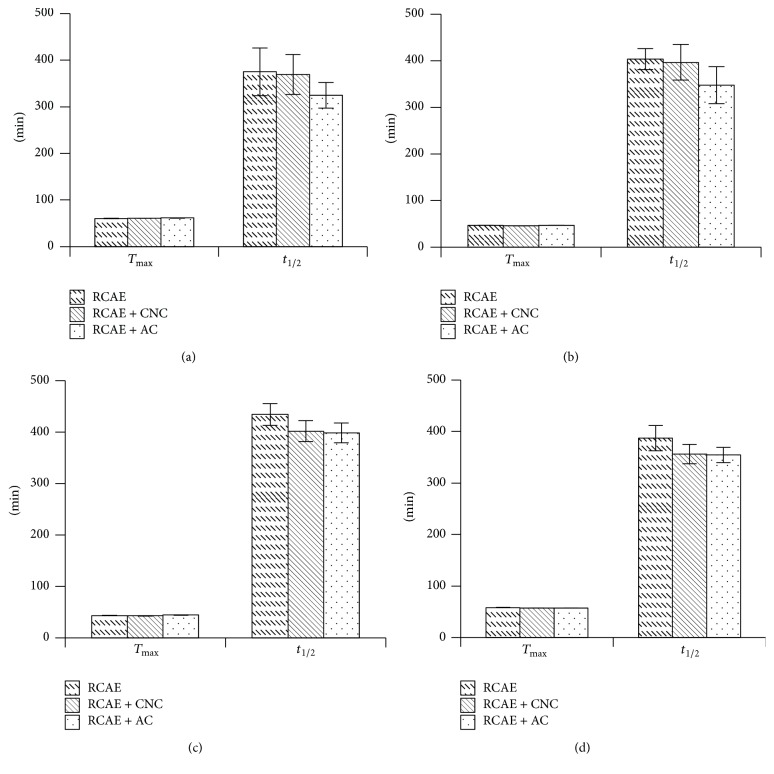
The time required to reach the maximum concentration (*T*_max_) and the half-life (*t*_1/2_) for berberine (a), coptisine (b), palmatine (c), and epiberberine (d).

**Figure 6:**
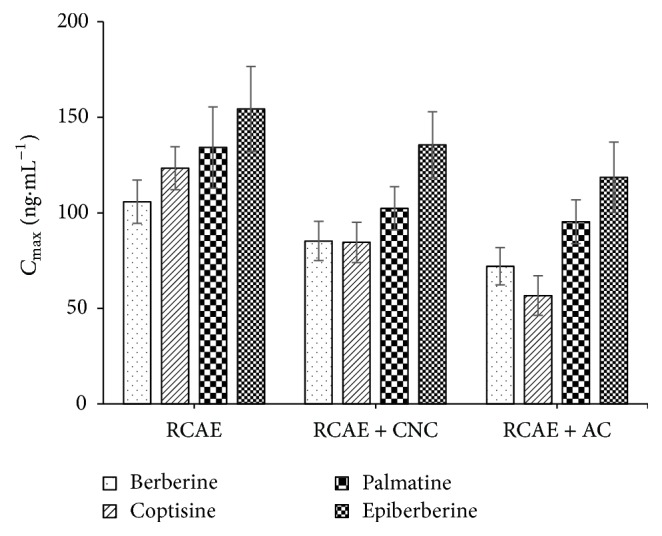
Maximum plasma concentration (*C*_max_) for berberine, coptisine, palmatine, and epiberberine.

Table 4 and Figures 5 and 6 showed the maximum plasma concentration (*C*_max_), the time required to reach the maximum concentration (*T*_max_), the area under the concentration-time curve (AUC_0–*t*_), and the half-life (*t*_1/2_) for berberine (Figure 5(a)), coptisine (Figure 5(b)), palmatine (Figure 5(c)), and epiberberine (Figure 5(d)).

There was a significant difference with regard to *C*_max_ of berberine between the groups, whereby Group A > Group B > Group C. However, there was no significant difference with regard to *T*_max_ among the three groups (*P* > 0.05). *t*_1/2_ of berberine in Group C was significantly lower than those of Groups A and B. The differences with regard to the AUC_0–*t*_ among the three groups were significant (*P* < 0.05), whereby Group B > Group A > Group C (Table 4). All of the above results (Figure 3(a)) indicated that CNC enhanced the bioavailability of berberine in comparison to AC, which decreased the bioavailability. The results also suggested that CNC may prolong the release of berberine in comparison to RCAE alone.

There was a significant difference with regard to *C*_max_ of coptisine among the groups, whereby Group A > Group B > Group C. However, there was no significant difference with regard to *T*_max_ among the three groups (*P* > 0.05). Meanwhile, *t*_1/2_ of coptisine for Group C was significantly lower than those of Groups A and B (*P* < 0.05). The difference with regard to the AUC_0–*t*_ among the three groups was significant (*P* < 0.05), with Group A > Group B > Group C (Table 4). All of the above results (Figure 3(b)) indicated that both CNC and AC decreased the bioavailability of coptisine in comparison to RCAE alone; however, CNC had a less significant effect compared to AC.

The *C*_max_ of palmatine in Group A was significantly higher than those of Groups B and C (*P* < 0.05), but the difference in *C*_max_ values between Groups B and C was not significant (*P* > 0.05). The differences with regard to *T*_max_ and *t*_1/2_ of palmatine among the three groups were not significant. Meanwhile, the AUC_0–*t*_ of Group A was higher than Groups B and C, whereby Group A > Group B > Group C (Table 4); however, the differences were not significant. These results (Figure 3(c)) indicated that both CNC and AC may decrease the bioavailability of palmatine in comparison to RCAE alone.

With regard to *C*_max_ of epiberberine, Group A showed a significantly higher concentration than Groups B and C (*P* < 0.05), but the difference between Groups B and C was not significant (*P* > 0.05). With regard to *T*_max_ and *t*_1/2_ of epiberberine, differences between the three groups were not significant. Furthermore, differences in the AUC_0–*t*_ between the groups were not significant (Table 3). These results indicated that CNC and AC may decrease the concentration of epiberberine in plasma in comparison to RCAE alone, although other parameters appear to be less affected.

## 4. Discussion

Carbonized herbal medicines have traditionally been used in Chinese medicine, with their use being first recorded 2000 years ago in* Prescriptions for Fifty-Two Diseases*. Recent researches have suggested that carbonized drugs may indeed have clinically relevant, curative effects [9–11]. However, investigation of the mechanism of action for carbonized Chinese herbal medicines has largely fallen behind their clinical applications and has mainly focused on the various chemical components and trace elements contained therein [12–15]. To address this shortfall, in this study the mechanism of carbonized herbal medicines was investigated via the effects of CNC on the pharmacokinetics of berberine, coptisine, palmatine, and epiberberine* in vivo*, which are the main alkaloids contained in RC. The results indicated that orally administered CNC in combination with RCAE enhanced the bioavailability of the alkaloids in comparison to RCAE and AC, and CNC prolonged the release of berberine in comparison to RCAE alone. However, CNC with RCAE resulted in decreased bioavailability of coptisine, palmatine, and epiberberine. The reason why CNC enhanced the bioavailability of some compounds over AC and prolonged the release of berberine may be due to the presence of CNC micropowder, which may adsorb alkaloids, thus prolonging their retention in the small intestine, from where they can be reabsorbed. However, this adsorption capacity may be strong, resulting in the decreased release of some alkaloids from CNC prior to excretion. This mechanism would account for the differences found here between the alkaloids as some may adhere more strongly to the micropowdered CS, hindering their release in the small intestine.

The bioavailability of berberine, coptisine, palmatine, and epiberberine decreased when RCAE was orally administered with AC. This result is in accordance with the accelerated clearance of drugs as a result of AC, with a concomitant decline of bioavailability [16, 17]. This is one of the reasons why AC has been used for the treatment of intoxication as a result of some drugs [7, 18]. However, the reason for the second peak in the concentration-time profiles of berberine and coptisine for Group C (Figure 2) is not well understood. However, similar secondary peaks have been observed in the concentration-time profiles of aconitine, hypaconitine, and mesaconine following administration of prepared Radix Glycyrrhizae and prepared* Aconitum carmichaelii* Debx. [19]. Typically, there are five reasons for a double peak concentration-time profile in pharmacology. Firstly, the drug may arrive at the small intestine in two (or more) batches due to nonuniform gastric emptying. The second reason may be that two different parts of the gastrointestinal tract are involved in drug absorption with different rates. The third possible explanation is due to the enterohepatic cycle. The fourth reason relates to pharmaceutics containing ingredients that delay release or promote fast release, and the fifth and final reason is due to the liposolubility of the drug distributed throughout the tissue, which may allow release of the drug into the blood again when the component in blood has declined to a certain extent. In this study the double peak concentration-time profile for some alkaloids in Group C may arise mainly as a result of pharmaceutic agents, with the aqueous extract providing fast release while the addition of AC resulted in delayed release.

## 5. Conclusions

The results of this research have shown that there was a significant difference between the effect of AC and CNC on the transportation of RC alkaloids* in vivo*, whereby AC results in a double peak concentration-time profile for some alkaloids. It was noteworthy that while CNC decreased the bioavailability of RC alkaloids in comparison to RCAE administered alone, it increased their bioavailability in comparison to AC and it prolonged the release of berberine.

Further investigations will be required to elucidate the precise mechanism of action of carbonized Chinese herbal medicines* in vivo*.

## Figures and Tables

**Table 1:** Calibration curves, correlation coefficients (*r*), and linear ranges of the four analytes in RCAE.

Compound	Calibration curve	*r* value	Linear range (ng/mL^−1^)
Epiberberine	*y* = 178.99*x* + 2.6353	0.9992	5.0–100
Coptisine	*y* = 508.97*x* + 3.9844	0.9990	1.4–140
Palmatine	*y* = 368.91*x* − 5.9279	0.9950	13.5–550
Berberine	*y* = 332.46*x* − 0.2947	0.9997	7.0–140

**Table 2:** Extraction replicates of the four analytes.

Compound	Origin amount/ng	Addition amount/ng	Measured amount/ng	Recovery rate (%)	Average recovery rate (%) (RSD%)
Epiberberine	50	25.0	74.3	97.2	98.5 (2.3)
	50	25.0	74.8	99.2	
	50	25.0	75.3	101.2	
	50	25.0	73.8	95.2	
	50	25.0	74.1	96.4	
	50	25.0	75.5	102.0	

Coptisine	35	17.5	52.4	99.4	98.0 (2.04)
	35	17.5	51.8	96.0	
	35	17.5	52.3	98.9	
	35	17.5	51.5	94.3	
	35	17.5	52.8	101.7	
	35	17.5	52.1	97.7	

Palmatine	55	27.5	81.1	94.9	96.2 (1.51)
	55	27.5	81.6	96.7	
	55	27.5	82.1	98.5	
	55	27.5	81.9	97.8	
	55	27.5	80.8	93.8	
	55	27.5	81.3	96.7	

Berberine	35	17.5	51.9	96.5	97.1 (0.98)
	35	17.5	52.1	97.7	
	35	17.5	51.7	95.4	
	35	17.5	52.1	97.7	
	35	17.5	51.9	96.6	
	35	17.5	52.3	98.8	

**Table 3:** Accuracy and precision of the four analytes in RCAE (*n* = 6).

Compound	Concentration (ng/mL)	Intraday precision			Interday precision		
		Measured amount ($\bar{x}$ ± *s*)	RSD (%)	RE (%)	Measured amount ($\bar{x}$ ± *s*)	RSD (%)	RE (%)
Coptisine	2.0	1.98 ± 0.033	0.41	1.0	1.97 ± 0.33	1.61	1.5
	35	34.950 ± 0.122	0.35	0.14	35.017 ± 0.213	0.61	0.048
	110	109.667 ± 0.314	0.28	0.30	109.967 ± 0.829	0.75	0.03

Berberine	10.0	9.932 ± 0.073	0.74	0.68	9.968 ± 0.117	1.17	0.032
	50.0	49.968 ± 0.084	0.17	0.11	50.050 ± 0.139	0.28	0.10
	110.0	109.93 ± 0.125	0.11	0.064	110.110 ± 0.827	0. 75	0.12

Palmatine	16	15.888 ± 0.085	0.53	0.70	15.922 ± 0.164	1.03	0.49
	200	199.682 ± 0.226	0.11	0.17	200.328 ± 0.839	0.42	0.164
	400	397.182 ± 1.594	0.40	0.71	399.945 ± 2.468	0.62	0.013

Epiberberine	6.0	5.96 ± 0.025	0.42	0.68	6.02 ± 0.052	0.87	0.33
	50.0	49.785 ± 0.093	0.19	0.43	50.047 ± 0.592	1.18	0.094
	80.0	79.713 ± 0.294	0.37	0.36	79.823 ± 0.356	0.45	0.22

**Table 4:** Area under concentration-time curve (AUC_0–*t*_) for the four analytes in Groups A–C.

Group	Berberine	Coptisine	Palmatine	Epiberberine
Group A	8123.2 ± 1734.1^b^	8092.3 ± 1423.7^a^	8674.3 ± 1534.7^a^	8415.1 ± 1434.2^a^
Group B	8432.21 ± 1831.3^a^	7532.4 ± 1231.4^b^	8347.2 ± 1617.4^b^	8117.4 ± 1534.3^b^
Group C	5472.41 ± 1041.7^c^	5317.3 ± 1047.4^c^	8274.7 ± 1537.8^b^	8074.2 ± 1374.5^b^

Note: “a, b, and c” indicated the difference of AUC_0–*t*_ among three groups.
